# The complete mitogenome of a peanut worm *Phascolosoma pacificum* (Sipuncula, Phascolosomatida, Phascolosomatidea)

**DOI:** 10.1080/23802359.2017.1372724

**Published:** 2017-09-05

**Authors:** Jin-Mo Sung, Mustafa Zafer Karagozlu, Chang-Bae Kim

**Affiliations:** Department of Biomaterial Engineering, Sangmyung University, Seoul, Korea

**Keywords:** Sipuncula, Phascolosomatida, Phascolosomatidea, complete mitogenome, *Phascolosoma pacificum*

## Abstract

Sipuncula (peanut worms) is a traditional phylum which consists of unsegmented bilateral symmetric marine worms. Although it is accepted as a phylum, the phylogenetic position of Sipuncula has been questionable. There is lack of molecular record on Sipuncula species while morphological data is not enough to distinguish the closer relationships between Annelida and Sipuncula. In order to add more data to genomic library of sipunculan species, *Phascolosoma pacificum* (Keferstein, 1866) was collected from seagrass area of Chuuk lagoon/Micronesia and its complete mitochondrial genome sequenced. Furthermore, phylogenetic relationship of the phylum Sipuncula and the other Trochozoan phyla investigate due to mitochondrial protein coding genes. Although there is lack of recorded data, our results support the idea that Sipuncula are nested within Annelida according to phylogenetic analyses of mitochondrial protein coding genes.

Sipunculans are spiralian unsegmented marine worms with 147 species and 17 genera (Schulze et al. 2005). The majority of species are relatively large and more than 50% of the species live in shallow-water inhabitants which makes them easy to collect and study. Moreover, 25 of 147 species belong to the genus *Phascolosoma* which are well characterized morphologically (Saiz et al. 2014; Johnson and Schulze 2016; WoRMS Editorial Board 2017). However, morphological similarity within the Sipuncula inhibits the developing phylogenetically informative characters for cladistics analyses (Schulze et al. 2005). In this content, analysing complete mitogenome might be useful to overcome these difficulties. However, there are only two complete mitogenomes recorded from the genus *Phascolosoma* which are *P. esculenta* (Shen et al. 2009) and *Phascolosoma* sp. (Sung et al. 2017). In order to improve the insufficient genomic studies, complete mitochondrial genome of *P. pacificum* is analysed and reported. This is the third complete mitogenome record of the genus.

The specimen has been collected from the sea grass area of Weno Island, Chook Lagoon, Federated States of Micronesia (7°26′41.0″N 151°53′57.7″E) on February 2015 and identified morphologically. It has been deposited in the Marine Biodiversity Institute of Korea (MABIK IV00155507) with 97% ethanol preservation. Analysing mitogenome and reconstruction of phylogenetic tree have been described in our previous study (Karagozlu et al. 2016).

Mitochondrial genome of *P. pacificum* was 16,039 bp in length (GenBank accession number KU820989). In comparison within the genus, mitogenome size of *P. pacificum* was in between *P. esculenta* (15,494 bp) and *Phascolosoma* sp. (16,571 bp) and the gene order was similar with the other records. The complete mitochondrial genome was composed of 13 protein coding genes, two ribosomal RNA genes, and 23 tRNA genes. All the genes were located on the major strand and the distribution of the mitogenome was 69% protein coding genes, 11.8% rRNA genes, 9.3% tRNA genes, and 19.9% non-coding area. The longest intergenic sequence was located between tRNA-Gly and tRNA-Val (1,203 bp). This region was A-T rich region and most probably it contains control region.

Trochozoa is a clade of Invertebrata that has unique larval development stage. This group consists of Sipuncula, Mollusca, Annelida, Nemertea, and Brachiopoda phyla. Although several studies focused on Trochozoan, the phylogenetic relationships of Sipuncula and Annelida are not confirmed (Bleidorn 2008; Hausdorf et al. 2010). Due to reconstructed phylogenetic tree, *Phascolosoma* sp. is the closest species to *P. pacificum* (Figure 1). The pairwise distance between two species is 0.16 while same value is 0.951 between *P. pacificum* and outgroup species. The other phascolosoma species *P. esculenta* has the same distance to *P. pacificum* and *Phascolosoma sp* (0.26). The monophyly of Phascolosomatidae was decelerated previously by nuclear and mitochondrial markers based study (Kawauchi et al. 2012; Sung et al. 2017). The phylogenetic tree also showed that there are some Annelida species which were early branched than sipunculans. The close relationships of these two groups were mentioned by previous study (Shen et al. 2009). This study provides additional data for molecular systematic studies of the genus *Phascolosoma*.

**Figure 1.**
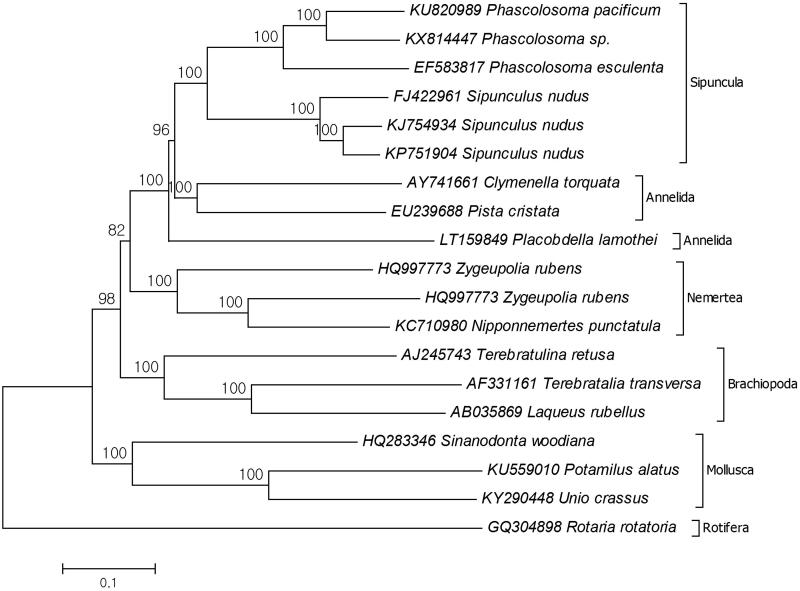
Reconstructed phylogenetic tree of *Phascolosoma pacificum* (KU820989). Three individual species from every Trochozoa phyla selected and their records retrieved from the GenBank. The species belongs to the phylum Rotifera selected as the representative of outgroup.
